# Peripheral blood and neuropsychological markers for the onset of action of antidepressant drugs in patients with Major Depressive Disorder

**DOI:** 10.1186/1471-244X-11-16

**Published:** 2011-01-26

**Authors:** André Tadić, Stefanie Wagner, Stanislav Gorbulev, Norbert Dahmen, Christoph Hiemke, Dieter F Braus, Klaus Lieb

**Affiliations:** 1 Department of Psychiatry and Psychotherapy, University Medical Centre, Mainz, Germany; 2 Interdisciplinary Centre for Clinical Trials (IZKS), University Medical Centre, Mainz, Germany; 3 Clinic for Psychiatry and Psychotherapy, Katzenelnbogen, Germany; 4 Clinic for Psychiatry and Psychotherapy, Dr. Horst-Schmidt-Kliniken, Wiesbaden, Germany

## Abstract

**Background:**

In Major Depressive Disorder (MDD), treatment outcomes with currently available strategies are often disappointing. Therefore, it is sensible to develop new strategies to increase remission rates in acutely depressed patients. Many studies reported that true drug response can be observed within 14 days (early improvement) of antidepressant treatment. The identical time course of symptom amelioration after early improvement in patients treated with antidepressants of all classes or with placebo strongly suggests a common biological mechanism, which is not specific for a particular antidepressant medication. However, the biology underlying early improvement and final treatment response is not understood and there is no established biological marker as yet, which can predict treatment response for the individual patient before initiation or during the course of antidepressant treatment. Peripheral blood markers and executive functions are particularly promising candidates as markers for the onset of action and thus the prediction of final treatment outcome in MDD.

**Methods/Design:**

The present paper presents the rationales, objectives and methods of a multi-centre study applying close-meshed repetitive measurements of peripheral blood and neuropsychological parameters in patients with MDD and healthy controls during a study period of eight weeks for the identification of biomarkers for the onset of antidepressants' action in patients with MDD. Peripheral blood parameters and depression severity are assessed in weekly intervals from baseline to week 8, executive performance in bi-weekly intervals. Patients are participating in a randomized controlled multi-level clinical trial, healthy controls are matched according to mean age, sex and general intelligence.

**Discussion:**

This investigation will help to identify a biomarker or a set of biomarkers with decision-making quality in the treatment of MDD in order to increase the currently disappointing remission rates of antidepressant treatment.

**Trial Registration:**

ClinicalTrials.gov: NCT00974155

## Background

Major depressive disorder (MDD) is a severe psychiatric disease that is characterized by depressed mood and loss of interest or pleasure in daily activities, and is accompanied by weight change, sleep disturbance, fatigue, physical impairment, diminished ability to think or concentrate and a high suicide rate. In Europe [1] and the United States (US) [2], MDD belongs to the most prevalent mental disorders with lifetime and 12-months prevalence rates in the total population as high as 12.8% (US: 16.2%) and 3.9% (US: 6.6%), respectively. Nearly all patients with MDD suffer from mild to very severe impairment in several domains of life like physical and social activities, or occupational responsibilities [3]. MDD produces substantial costs through hospital admissions, outpatient care and productivity loss as a result of depression-related morbidity, suicide, and other relevant parameters [4,5].

### Treatment outcome of MDD

The above mentioned data clearly indicate the utmost importance of effective treatments for MDD. The use of antidepressant drugs (ADs) for the treatment of MDD is well established. However, effect sizes of currently available antidepressants are rather small than medium [6-8] and treatment outcome remains disappointing with remission rates of maximal 37% [[9] and references inside]. Hence, it is sensible to develop new strategies to increase remission rates in acutely depressed patients.

### Onset of antidepressants' action

For decades, it has been common clinical view that antidepressant response appears with a delay of several weeks [10,11]. This hypothesis of a delayed action of ADs had substantial impact on clinical practice. The recommended treatment duration until insufficient outcome can be assumed and treatment should be optimised ranges between 3-4 weeks [12,13] and 4-8 weeks [14]. As marker for onset of action, a symptom reduction of ≥ 50% at week 4 compared to treatment initiation is generally accepted. Challenging the idea of a delayed onset of ADs' action, there is a substantial body of evidence from many retrospective studies with more than 33.000 patients treated with virtually all groups of ADs strongly suggesting that a true drug response can be observed within the first 10-14 days of treatment [15-25]. The occurrence of improvement of depressive symptoms in the early course of treatment has been identified as being highly predictive for final treatment outcome [15-18,22-25], corroborating the idea that early improvement (typically defined as a 20% reduction of depressive symptoms, measured with rating scales like the Hamilton Depression Rating Scale) is an important clinical model for the onset of antidepressants' action [26].

### Biology underlying early improvement

Furthermore, it resulted in the idea that an effective antidepressant treatment triggers and maintains conditions necessary for recovery from the disorder. It has been suggested that a biological, "resilience"-like component is possessed that controls recovery from depression to a major extent. Once triggered, recovery seems to follow independent of pharmacologic differences of the triggers. Consequently, the vast majority of patients showing a favourable later outcome experience the respective onset within the first 2 weeks of treatment. Inversely, non-improvement after 2 weeks of treatment seems to indicate that a selected AD did not trigger the resilience-like component and has strongly limited chances to do so, even if continued in the course of treatment [22].

### Biomarkers could establish the basis for individualised treatment approaches

The virtually identical time course of symptom amelioration after early improvement in patients treated with antidepressants of all classes or with placebo strongly suggests that early improvement and the successive time course of response reflect a common biological mechanism, which is not specific for a particular antidepressant medication. However, the neurobiological substrates of this remarkably robust relation between *early improvement *and *final treatment outcome *need to be elucidated. The lack of this knowledge also means that there is currently no validated biomarker for the onset of antidepressants' action and final treatment response during the course or before the initiation of an antidepressant treatment. The identification of biomarkers could lead to the development of effective personalized antidepressant treatment. Biomarkers may give an insight into the underlying biological basis of depression, which can be used to develop more effective drug treatments and therefore shorten the time to response or remission. The term 'biomarker' is used here to describe a biological change associated with depression that could be used to indicate the presence and severity of the condition and predict drug or other treatments' response as well as the clinical prognosis [27]. The idea behind identifying biomarkers is that they will allow the identification of patients that benefit from antidepressants that specifically target a patient's individual psychopathology [28]. The present scientific investigation should help to close this significant gap of current research and lead to the identification of biomarkers that increase the risk for depression and mirror the antidepressant treatment response. Thereby, these analyses should establish the basis for individualised treatment approaches, leading to better treatment outcomes with less adverse effects and in a shorter period of time.

### Peripheral blood markers could serve as biomarkers for antidepressant treatment response

Although the search for peripheral blood markers for psychiatric disorders lasts for many years, a non-invasive blood-based test that could be used for diagnosis, help to stratify patients based on disease subtypes or indicating the onset of antidepressants' action has not been identified as yet. Several neurotrophic factors comprising brain- and glia-derived neurotrophic factors as well as cytokines and insulin-like growth factor 1 are discussed as potential blood markers [27]. For depression, monoamine-related markers have been studied with only partial success in terms of specificity of the marker, or replication of the findings. More recently, a number of studies have been carried out to evaluate the potential of neurotrophic markers such as the brain derived neurotrophic factor (BDNF) in different psychiatric diseases, again resulting in evidence of association but also with many non specific or conflicting findings. The finding of an HPA dysfunction in depressed patients during acute phase has led to the development of neuro-endocrine challenge tests as putative biomarkers. Another interesting line of research has focused on inflammatory-related markers, based on the evidence of reciprocal communication between immune and nervous systems and of altered immunological state in psychiatric diseases. For depression in particular a ''cytokine hypothesis'' has been developed that associates the dysregulation of the immuno-inflammatory system with the aetiology and the pathophysiology of MDD. Recently, a larger panel of pro- and anti-inflammatory cytokines was measured in a case/control population of MDD showing elevation of a number of additional cytokines not previously implicated in MDD, as well as of some previously untested chemokines [29].

In the context of personalized medicine, it might be straightforward to identify valid biomarkers based on protein analysis, because most drugs act on proteins. However, such biomarkers remain to be discovered; the human body is believed to contain more than a million different proteins and their expression fluctuates constantly [28]. Another reason reason for the failure of previous studies on biomarkers for the onset of antidepressants' action might be that they were usually restricted to either one measurement (baseline) or two measurements with an interval of at least 4 weeks (e.g. before and after antidepressant treatment), reflecting the traditional view of a delayed onset of antidepressants' action. Taking into account the above mentioned studies showing that true drug response can be observed within the first 10-14 days of antidepressant treatment, it might be more appropriate and promising to focus on biomarkers' reactions in the first 7-14 days after initiation of antidepressant treatment.

### Executive functions could serve as markers for antidepressant treatment outcome

A further approach to identify markers of treatment response in Major Depressive Disorder (MDD) is the investigation of neuropsychological functions. In patients with MDD, empirical evidence supports the existence of moderate but significant neuropsychological deficits [30,31]. With respect to cognitive domains, impairment has been reported for executive functioning in particular [32], whereas less significant deficits have been found for psychomotor speed [33], attention [34] and memory [35]. Deficits seem to increase with the number of depressive episodes, melancholic symptoms and age [36,37]. Many studies reported a substantial improvement in neuropsychological functioning during the course of an antidepressant treatment in patients with MDD [30,37-39]. Nevertheless, the results of these studies are heterogeneous and support the hypothesis that some cognitive deficits, like executive dysfunctions, improve during the course of an antidepressant treatment whereas other impairments, specifically memory impairments, often persist after the remission of the depressive symptoms [40,41]. Multiple studies reported an association between the time course of symptom amelioration of MDD and the performance in word fluency, cognitive flexibility and working memory tasks [31,38,39,42-45]. Furthermore, studies show that non-responders to an antidepressant treatment have a poorer pre-treatment performance in cognitive functions than responders [46].

Recent studies demonstrated the involvement of a consistent set of limbic and cortical regions in both unipolar and bipolar depression as well as replicable patterns of activation changes with various antidepressant treatments [47,48]. Furthermore, a fluoxetine study in patients with MDD revealed sub-cortical metabolic changes, which were already seen after 1 week of antidepressant treatment, although patients showed no change in depressive symptoms. The reversal of this week-1 pattern at 6 weeks was seen uniquely in those patients showing a clinical response. These results suggest a requisite process of neural adaptation in specific brain regions during antidepressant treatment [47,48].

### Objectives

The present paper presents the rationales, objectives and methods of two complementing clinical studies [additional scientific investigations to the "Randomised clinical trial comparing early medication change (EMC) strategy with treatment as usual (TAU) in patients with Major Depressive Disorder - the EMC trial" and "the EMC Control study"] applying repetitive measurements of peripheral blood and neuropsychological parameters in patients with MDD and healthy controls during a period of eight weeks in order to identify biomarkers for the onset of antidepressants' action in patients with MDD.

Previous studies suggest that peripheral blood and neuropsychological parameters might be useful in the prediction of treatment response before and after initiation of an antidepressant treatment and thus might be useful for the selection of a particular antidepressant medication. Therefore, the present study has two main objectives:

*I. Association between early changes of peripheral blood parameter/neuropsychological functioning with final treatment outcome*:

▪ Changes of peripheral blood parameters/neuropsychological functioning in the early course of treatment (baseline [BL] - day 7/14) account for a high percentage of the variance of final changes of depression severity (HAMD-17).

▪ Changes of peripheral blood parameters/neuropsychological functioning in the early course of treatment (baseline [BL] - day 7/14) predict later treatment response and remission with high sensitivity and specificity.

*II. Association between a concurrent occurrence of early changes of peripheral blood parameters/neuropsychological function and early improvement with final treatment outcome*:

▪ Concurrent changes of peripheral blood parameters/neuropsychological functioning plus early improvement account for a higher percentage of variance of final changes in depression severity than early changes of peripheral blood markers or early improvement alone.

▪ Concurrent changes of peripheral blood parameters/neuropsychological functioning plus early improvement predicted later treatment response or remission with higher sensitivity and specificity than early changes of peripheral blood parameters or early improvement alone.

## Methods/Design

### Participants

#### Patients

In line with the above mentioned rationales and objectives, the herein presented study in patients was designed as a scientific investigation additional to the "Randomised clinical trial comparing an early medication change (EMC) strategy with treatment as usual (TAU) in patients with Major Depressive Disorders (MDD) - The EMC Trial (ClinicalTrials.gov identifier n°: NCT00974155)". The detailed study protocol of The EMC Trial has been reported previously [49]. In brief, The EMC Trial is a phase IV, multi-centre, multi-step, randomized, observer-blinded, actively controlled parallel-group clinical trial to investigate for the first time prospectively, whether non-improvers after 14 days of antidepressant treatment with an early medication change (EMC) are more likely to attain remission (HAMD-17 ≤ 7) on treatment day 56 compared to patients treated according to current guideline recommendation (treatment as usual; TAU). In level 1 of the EMC trial, non-improvers after 14 days of antidepressant treatment will be randomised to an EMC strategy or TAU. The EMC strategy for this study schedules a first medication change on day 15; in case of non-improvement between days 15-28, a second medication change will be performed. TAU schedules the first medication change after 28 days in case of non-response (HAMD-17 decrease <50%). Both interventions will last 42 days. In levels 2 and 3, EMC strategies will be compared with TAU strategies in improvers on day 14, who experience a stagnation of improvement during the course of treatment. The trial is supported by the German Federal Ministry of Education and Research (BMBF) and is conducted in cooperation with the BMBF funded Interdisciplinary Centre for Clinical Trials (IZKS) at the University Medical Centre Mainz and at six clinical trial sites in Germany.

In order to acquire a sample representative of inpatients with MDD, the study has broad inclusion criteria that allow enrolment of both adult and elderly patients, moderately to very severely depressed patients as well as MDD patients with psychiatric comorbid disorders. The detailed in- and exclusion criteria have been previously reported [49]. Key inclusion criteria are [1] Major Depressive Disorder (MDD), first episode or recurrent, according to DSM-IV; [2] a HAMD17 score of ≥18 pts.; [3] age 18 - 65 years and ≤60 years at the time of the first depressive episode. Key exclusion criteria are [1] acute risk of suicide needing an intervention not comprised by protocol treatment (e.g. electroconvulsive therapy); [2] lifetime DSM-IV diagnosis of dementia, schizophrenia, schizoaffective disorder, bipolar disorder; [3] current DSM-IV diagnosis of posttraumatic stress disorder, obsessive-compulsive disorder, anxiety disorder, or eating disorder and the requirement of a treatment not comprised by protocol treatment; [4] DSM-IV substance dependency requiring acute detoxification; [5] depression due to organic brain disorder, e.g. Multiple Sclerosis and Parkinson's Disease; [6] women who are pregnant, breastfeeding or planning to become pregnant during the trial.

#### Healthy volunteers

In order to assure the specificity of the study results, the results of MDD patients will be compared to those of healthy controls. Seventy-five healthy controls will be included in the study. Patients and healthy controls will be matched by age, gender and general intelligence. The inclusion criteria are: [1] mentally healthy, confirmed by the M.I.NI. International Neuropsychiatric Interview and the Structured Clinical Interview for DSM-IV Axis II Personality Disorders (SCID-II); [2] ability of subjects to understand character and individual consequences of clinical trial; [3] signed and dated informed consent of the subject must be available before start of any specific trial procedures. The exclusion criteria are: [1] current medication; [2] missing German language ability; [3] cognitive impairment which interferes with subjects' ability to participate in the psychopathological interviews or neuropsychological testing; [4] a history of cranio-cerebral injury; [5] relevant organic disease, e.g. Multiple Sclerosis or Morbus Parkinson.

### Study procedures

Table 1 shows the study procedures for patients and healthy controls.

**Table 1:** Trial schedule of patients and healthy controls

Visit (V)/Action	SC	BL	V1	V2	V3	V4	V5	V6	V7	V8
**Trial day**	**-14-0**	**0**	**7 ± 2**	**14 ± 2**	**21 ± 2**	**28 ± 2**	**35 ± 2**	**42 ± 2**	**49 ± 2**	**56 ± 2**

**Prescreening**										

DIA-X-SSQ ^1)^	X									

**Basic documentation**										

Inclusion/exclusion criteria		X								

Patient information and consent		X								

Demographics		X								

Medical history		X								

**Diagnostic procedures**										

M.I.N.I. SCID-II	X^2) ^X	X^3) ^X								

**Treatment outcome**										

HAMD-17 IDS-C30 IDS-SR30 SF-12		X	X	X	X	X	X	X	X	X

**Peripheral blood**										

Serum		X	X	X	X	X	X	X	X	X

Plasma		X	X	X	X	X	X	X	X	X

Whole blood (EDTA)		X	X	X	X	X	X	X	X	X

**Neuropsychology**										

MWT		X								

RWT		X		X		X		X		X

TMT A/B		X		X		X		X		X

RWT (category change)		X		X						X

DOT		X		X						X

RFFT		X		X						X

**End of trial**										X

1) only in healthy controls; 2) in patients at screening visit; 3) in healthy controls at baseline visit; SC = Screening; BL = Baseline; V1 = Visit 1, V2 = Visit 2, V3 = Visit 3, V4 = Visit 4, V5 = Visit 5, V6 = Visit 6, V7 = Visit 7, V8 = Visit 8; DIA-X-SSQ = Screening Questionnaire of the DIA-X-Interview, M.I.N.I. = MINI International Neuropsychiatric Interview; SCID-II = Structured Clinical Interview for DSM-IV Axis II Personality Disorders; HAMD17 = Hamilton Depression Rating Scale; IDS-C30 = Inventory of Depressive Symptoms - Interview, IDS-SR30 = Inventory of Depressive Symptoms - Self rating, MWT = Multiple Vocabulary Test, TMT = Trail Making Test, DOT = Adaptive Digit Ordering Test, RFFT = Ruff Figural Fluency Test

#### Assessment of mental disorders

Diagnosis as well as possible comorbid psychiatric diseases will be assessed at screening (EMC Trial) or at baseline (controls).

• *DIA-X-SSQ *[50]: For the pre-screening of DSM-IV axis I disorders in healthy controls, the screening questionnaire of the DIA-X-Interview will be applied.

• *M.I.N.I. International Neuropsychiatric Interview *[51]: The M.I.N.I is a structured clinical interview to assess mental disorders according to DSM-IV [52] and ICD-19 [53].

• *Structured Clinical Interview for DSM-IV Axis II Personality Disorders (Scid-II) *[54]: The SCID is a structured clinical interview to diagnose personality disorders.

#### Assessment of depression severity

• *Hamilton Depression Rating Scale (*HAMD17) [55]: Depression severity will be assessed using the 17-item version of the Hamilton-Depression-Rating-Scale (HAMD17). Each item refers to a different depressive symptom; the severity of each symptom will be expressed with a score ranging from 0-2, 0-3, or 0-4.

• *Inventory of Depressive Symptoms (*IDS-C30/-SR30) [56]: Additionally, depression severity will be assessed with the 30-items clinician-rated and self-rated version of the Inventory of Depressive Symptomatology (IDS-C30 and IDS-SR30, resp.). Each item refers to a different depressive symptom; the severity of each symptom will be expressed with a score ranging from 0-3.

#### Assessment of function

• *Short-Form Health Survey *(SF-12) [57]: The SF-12 is a measure for health-related quality of life independent of psychiatric diagnosis. Its 12-item version assesses the two dimensions "physical health" and "psychic health" as subscales. Each item refers to a different symptom concerning "physical health" and "psychic health"; the severity of each symptom will be expressed with a score ranging from 1-2, 1-3, 1-5, or 1-6.

#### Biomaterial

• *Serum/plasma*: For the purpose of identification of serum and plasma markers of depression and antidepressant treatment response, serum and plasma proteins, which are possibly suitable to discriminate between depressive patients and healthy controls or to predict treatment response in major depression, will be analyzed in parallel to clinical assessments, i.e. from baseline to day 56 in weekly intervals. Serum and plasma will be extracted from whole blood using standard laboratory procedures and deep frozen (-80°C) until analysis. Date and time of blood withdrawal, time of start and stop of centrifugation as well as time of placement in the freezer will be recorded in the electronic case record form (eCRF).

• *Molecular genetic markers*: For the purpose of identification of molecular genetic markers of depression and antidepressant treatment response, molecular genetic markers (DNA variations, epigenetic structures, RNA expression), which are possibly suitable to discriminate between depressive patients and healthy controls or to predict treatment response in major depression will be analyzed. For these analyses, whole blood (EDTA) samples at the baseline visit and at each following visit (V1-8) are necessary. Whole blood will be deep frozen until analysis. Date and time of blood withdrawal as well as the time of placement in the freezer (-80°C) will be recorded in the eCRF.

#### Neuropsychological tests

• *Multiple Vocabulary Test *(MVT) [58]: Premorbid intelligence is examined using a test for crystallized intelligence. Patients have to differentiate real German words from pseudowords. Results are reported as the raw score of the correct words. The MVT will be applied at baseline.

• *Verbal fluency Test (*RWT) [59]: Verbal fluency is assessed by the Regensburger Verbal Fluency Test (RWT). The RWT is composed of lexical and semantic fluency tasks. In the subtest "Verbal Letter Fluency", participants will be instructed to generate as many words beginning with a specific letter as they could think of in 2 minutes. In the semantic fluency task, subjects will be instructed to generate as many words (e.g. dog) as possibly being part of a specific category (e.g. animals) in 2 minutes. The measure of performance is the number of correct words given in 2 minutes. The RWT consists of five alternate forms. Each of the alternate forms will be applied once at baseline and then in bi-weekly intervals (visits 2, 4, 6, and 8). The five alternate versions were randomly distributed to the visits.

• *Trail Making Test (*TMT) [60]: The TMT is a frequently used measure of executive cognitive functions. The TMT-A assesses processing speed, the TMT-B cognitive flexibility und task swifting. Both parts of the Trail Making Test consist of 25 circles distributed over a sheet of paper. In part A, the circles are numbered 1-25, and the patient should draw lines to connect the numbers in ascending order. In part B, the circles include both numbers (1-13) and letters (A-L); as in part A, the patient draws lines to connect the circles in an ascending pattern, but with the added task of alternating between the numbers and letters (i.e., 1-A-2-B-3-C, etc.). In a previous study we developed and validated three alternate forms of the TMT A and B (Wagner et al., in preperation). Thus, the TMT consists of four alternate forms, which were randomly distributed to the visits.

• *Adaptive Digit Ordering Test (*DOT) [61]: Working memory is assessed by the DOT. The DOT consists of six items of increasing length (three to eight digits). Each item comprises of two trials. Subjects are asked to repeat these digits in accenting order immediately after presentation. The DOT consists of two alternate forms. One alternate version will be applied at baseline and visit 8, the other version in visit 2. The versions were randomly distributed to the subjects.

• *Ruff Figural Fluency Test (RFFT) *[62]: The RFFT was developed to provide clinical information regarding nonverbal capacity for fluid and divergent thinking, ability to flexibly shift cognitive set, planning strategies, and executive ability to coordinate this process. The RFFT was designed as a nonverbal analogue to popular verbal fluency tests. The Test Booklet consists of five 60-second parts, each with a different stimulus presentation. The task is to draw as many unique designs as possible within a set period of time (60 seconds) by connecting the dots in different patterns. The RFFT is applied at baseline as well as in visite 2 and 8. It consists of five alternate forms. We use the alternate forms 1, 4 and 5 in this study, because version 4 and 5 are variations of the original version 1. The three versions were randomly distributed to the visits.

### Sample size

The sample size calculation is based on the large body of evidence showing a different treatment outcome in patients with or without early improvement as well as on the assumption of a close relationship between early improvement and a relevant change of biomarkers. For the sample size calculation we assume that patients with early improvement display specific changes of biomarkers and that treatment response will be higher in subjects with biomarker changes (group 1) than in patients without biomarker changes (group 2). Differences between the frequency of patients with and without these changes will be calculated by a Chi^2^-Test. Significance will be set at p ≤ 0.05. Based on treatment response rates of patients with or without early improvement we expect a treatment response rate of 0.5 in group 1 and of 0.2 in group 2. These proportions result in an odds ratio of 0.250. Expecting a sample size ratio between group 1 and 2 of 0.54, a required sample sizes of 128 patients per group (alpha = 0.05, Fisher's exact test, 2-sided) for a power of 80% will be needed. For the replication sample, the same sample size is assumed (=> 256 patients). If 12% of patients are dropouts, a total sample size of N = 287 patients will be needed.

Four of the six trial sites of the EMC Trial are involved in the collection of blood; at these trial sites, approximately 450 patients will be included during the study period. Neuropsychological functioning will be assessed at two trial sites of the EMC trial; at these trial sites, approximately 290 patients will be investigated. Therefore, the recruited number of patients will be sufficient for the testing of the above mentioned hypotheses including replication samples.

Differences between patients and healthy controls will be calculated by t-tests for independent variables (alpha = 0.05, 2-sided). For a power of 90% and an effect size of .80 a sample size of 50 healthy controls will be needed. Due to a lower motivation of the healthy controls to participate in the study during the whole study period, a drop out rate of 25% in healthy control is assumed. Therefore, it is planned to assess 70 healthy controls.

### Staff training

For the collection of biomaterial, standard operating procedures (SOP) have been developed and thoroughly tested at the Department of Psychiatry and Psychotherapy at the University Medical Center Mainz (UMCM). At each trial site, study nurses were trained in the application of SOPs by staff members of the Dept. of Psychiatry and Psychotherapy, UMCM. Study nurses of trial sites are supervised in monthly intervals.

For the assessment of depression severity, 17 psychologists and four residents in psychiatry were trained (HAMD-17; IDS). The training was carried out using five video tapes of patients with DSM-IV Major Depression [63]. The training revealed that accuracy and inter-rater reliability of the HAMD_17 _and IDS_C30 _were already high in the first rating and increased during the course of the training [64]. The training sessions were organized in a standardized manner. After an introduction section on the theoretic background and use of HAMD and IDS, five HAMD_17 _and three IDS_30CR _videos were shown. After each video, individual ratings were carried out following a discussion of the results.

Psychologists of the in the participating trial sites were trained in the application of the neuropsychological tests. At the beginning, all raters had to execute the test by themselves. After that, there was an introduction in theoretic background and test procedures of the neuropsychological tests. Last, all raters had to execute the tests under supervision of an expert in neuropsychological testing (SW).

### Ethical issues

The procedures set out in this trial protocol, pertaining to the conduct, evaluation, and documentation of this trial, are designed to ensure that all persons involved in the trial abide by good clinical practice (GCP) and the ethical principles described in the Declaration of Helsinki. The trial will be carried out in accordance with local legal and regulatory requirements. The requirements of the AMG, the GCP regulation, and the Federal Data Protection Law (BDSG) will be kept. Before being admitted to the clinical trial, the subject must consent to participate after being fully informed about the nature, scope, and possible consequences of the clinical trial. After reading the informed consent document, the subject must give consent in writing. The subject's consent must be confirmed by the personally dated signature of the subject and by the personally dated signature of the person conducting the informed consent discussions. All study components were approved by the local ethics committee of the Landesärztekammer Rheinland-Pfalz (study code n°: 837.211.09/6717 (patients), n°: 837.476.09/6982 (healthy controls)) and is compliant with the Code of Ethics of the World Medical Association (Declaration of Helsinki).

## Discussion

The traditional idea of a delayed onset of antidepressants' action was fundamental for the design of previous studies searching for biomarkers indicating the onset of antidepressants' action. As a consequence of the delayed-onset hypothesis, previous studies in the field mainly focused on treatment outcomes after several weeks or even months of antidepressant treatment. This approach might be related to the fact that there are no established biomarkers for the onset of antidepressants' action as yet and treatment efficacy is only determined by clinical measures and in the later course of treatment. Currently available data can not answer the question, whether the changes of peripheral blood or neuropsychological parameters during the course of treatment might be useful biomarkers in clinical practice or in the research of new antidepressant substances, because studies so far were typically restricted to two measurements, one before and one after antidepressant treatment. In order to evaluate the potential clinical value of measuring biomarker changes, it is essential to evaluate the significance of early changes of biomarkers for the finally achieved changes of these markers and, even more important, depression severity. For the investigation of the predictive value of early changes of biomarkers for final treatment outcome, repetitive measures in weekly intervals are necessary to demonstrate the detailed time course of biomarkers as well as depression severity. The present study is a scientific investigation conducting close-meshed repetitive measurements of peripheral blood and neuropsychological markers for the onset of antidepressants' action and final treatment response in patients with MDD (DSM-IV) during a period of eight weeks. The assessment of peripheral blood and executive parameters in patients participating in this study should extent the existing knowledge about their predictive value for antidepressant treatment response. These analyses should further broaden the basis for individualised treatment approaches, leading to better treatment outcomes with less adverse effects and in a shorter period of time. The present study is unique because it will enable for the first time the determination of a close-meshed time course of many peripheral blood and neuropsychological parameters in parallel to depression severity, taking into account the large data base showing that the onset of antidepressants' action takes place in the first 10-14 days after treatment initiation. Parallel to this close-meshed collection of biomaterial, detailed blinded assessments of psychopathology by trained raters establish the phenotype "treatment outcome in MD". The aim of this study is to investigate the relationship between ii) early changes of peripheral blood markers/neuropsychological performance and final changes of depression severity during short-term antidepressant treatment in patients with MDD; and ii) a concurrent occurrence of early changes of peripheral blood parameters/neuropsychological functioning plus early improvement with the final treatment outcome. With this multi-level investigation we hope to provide data helping to establish a biomarker or a set of biomarkers with decision-making quality in the treatment of MDD in order to increase the currently disappointing remission rates of antidepressant treatment. For this goal, external researchers may collaborate and request access to the material or data.

## List of abbreviations used

AD: antidepressant drug; AMG: Arzneimittelgesetz; BDNF: brain-derived neurotrophic factor; BDSG: Federal Data protection Law; BL: baseline; BMBF: German Federal Ministry for Education and Research; DFG: Deutsche Forschungsgesellschaft; DIA-X-SSQ: Diagnostic expert system for mental disease, Stamm Screening Questionnaire; DOT: Adaptive Digit Ordering Test; DSM-IV: Diagnostic and statistical Manual of mental disease; EMC: Early Medication Change; GCP: good clinical practice; HAMD: Hamilton Depression Rating Scale; IDS: Inventory of Depressive Symptoms; IZKS: Interdisciplinary Centre for Clinical Trials; MDD: Major Depressive Disorder; M.I.N.I.: MINI International Neuropsychatric Interview; MVT: Multiple Vocabulary Test; N: number; Pts: points; RFFT: Ruff Figural Fluency Test; RWT: Regensburger Wortflüssigkeitstest; SCID-II: Structured Clinical Interview for DSM-IV Axis II Personality Disorders; SF-12: Short-Form Health Survey; TAU: treatment as usual; TMT: Trail Making Test; US: United States

## Competing interests

The authors declare that they have no competing interests.

## Authors' contributions

AT, SW and KL developed the idea of the herein reported studies. AT, SW, ND, CH, DFB, KL participated in the conception and design of the trial. AT and SW wrote the study protocol. AT and SW drafted the manuscript. All authors critically read and approved the final version of the manuscript. The corresponding author had final responsibility for the decision to submit for publication.

## Pre-publication history

The pre-publication history for this paper can be accessed here:

http://www.biomedcentral.com/1471-244X/11/16/prepub

## References

[B1] Alonso J Angermeyer MC Bernert S Bruffaerts R Brugha TS Bryson H de Girolamo G Graaf R Demyttenaere K Gasquet I Haro JM Katz SJ Kessler RC Kovess V Lépine JP Ormel J Polidori G Russo LJ Vilagut G Almansa J Arbabzadeh-Bouchez S Autonell J Bernal M Buist-Bouwman MA Codony M Domingo-Salvany A Ferrer M Joo SS Martínez-Alonso M Matschinger H Mazzi F Morgan Z Morosini P Palacín C Romera B Taub N Vollebergh WA Prevalence of mental disorders in Europe: results from the European Study of the Epidemiology of Mental Disorders (ESEMeD) project Acta Psychiatrica Scandinavica 2004 109 Suppl. 420 21 27 10.1111/j.1600-0047.2004.00327.x 15128384

[B2] Kessler RC Berglund P Demler O Jin R Koretz D Merikangas KR Rush AJ Walters EE Wang PS National Comorbidity Survey Replication The epidemiology of major depressive disorder: results from the National Comorbidity Survey Replication (NCS-R) JAMA 2003 289 3095 3105 10.1001/jama.289.23.3095 12813115

[B3] Mathers CD Loncar D Projections of global mortality and burden of disease from 2002 to 2030 PLoS Med 2006 3 e442 10.1371/journal.pmed.0030442 17132052 PMC1664601

[B4] Sobocki P Jönsson B Angst J Rehnberg C Cost of depression in Europe Journal of Mental Health Policy Econ 2006 9 87 98 17007486

[B5] Greenberg PE Kessler RC Birnbaum HG Leong SA Lowe SW Berglund PA Corey-Lisle PK The economic burden of depression in the United States: how did it change between 1990 and 2000? Journal of Clinical Psychiatry 2003 64 1465 1475 10.4088/JCP.v64n1211 14728109

[B6] Turner EH Matthews AM Linardatos E Tell RA Rosenthal R Selective publication of antidepressant trials and its influence on apparent efficacy New England Journal of Medicine 2008 358 252 260 10.1056/NEJMsa065779 18199864

[B7] Kirsch I Deacon BJ Huedo-Medina TB Scoboria A Moore TJ Johnson BT Initial severity and antidepressant benefits: a meta-analysis of data submitted to the Food and Drug Administration PLoS Med 2008 5 45 10.1371/journal.pmed.0050045 PMC2253608 18303940

[B8] Fournier JC DeRubeis RJ Hollon SD Dimidjian S Amsterdam JD Shelton RC Fawcett J Antidepressant drug effects and depression severity: a patient-level meta-analysis JAMA 2010 303 47 53 10.1001/jama.2009.1943 20051569 PMC3712503

[B9] Warden D Rush AJ Trivedi MH Fava M Wisniewski SR The STAR*D Project results: a comprehensive review of findings Current Psychiatry Report 2007 9 449 459 10.1007/s11920-007-0061-3 18221624

[B10] Quitkin FM Rabkin JG Ross D Stewart JW Identification of true drug response to antidepressants. Use of pattern analysis Archives of General Psychiatry 1984 41 782 786 6378117 10.1001/archpsyc.1984.01790190056007

[B11] Quitkin FM Rabkin JD Markowitz JM Stewart JW McGrath PJ Harrison W Use of pattern analysis to identify true drug response. A replication Archives of General Psychiatry 1987 44 259 264 3548638 10.1001/archpsyc.1987.01800150071009

[B12] DGPPN, BÄK, KBV, AWMF, AkdÄ, BPtK, BApK, DAGSHG, DEGAM, DGPM, DGPs, DGRW (Hrsg) für die Leitliniengruppe Unipolare Depression S3-Leitlinie/Nationale Versorgungsleitlinie Unipolare Depression-Langfassung 2009 http://www.dgppn.de http://www.versorgungsleitlinien.de, http://www.awmf-leitlinien.de. 1. Auflage 2009. DGPPN, ÄZQ, AWMF-Berlin, Düsseldorf 2009. Internet

[B13] Bauer M Bschor T Pfennig A Whybrow PC Angst J Versiani M Möller HJ WFSBP Task Force on Unipolar Depressive Disorders. World Federation of Societies of Biological Psychiatry (WFSBP) Guidelines for Biological Treatment of Unipolar Depressive Disorders in Primary Care The World Journal of Biological Psychiatry 2007 8 67 104 10.1080/15622970701227829 17455102

[B14] American Psychiatric Association Practice guideline for the treatment of patients with major depressive disorder (revision) American Journal of Psychiatry 2000 157 Suppl. 4 1 45 10767867

[B15] Nierenberg AA Farabaugh AH Alpert JE Gordon J Worthington JJ Rosenbaum JF Fava M Timing of onset of antidepressant response with fluoxetine treatment American Journal of Psychiatry 2000 157 1423 1428 10.1176/appi.ajp.157.9.1423 10964858

[B16] Katz MM Tekell JL Bowden CL Brannan S Houston JP Berman N Frazer A Onset and early behavioral effects of pharmacologically different antidepressants and placebo in depression Neuropsychopharmacology 2004 29 566 579 10.1038/sj.npp.1300341 14627997

[B17] Henkel V Seemüller F Obermeier M Adli M Bauer M Mundt C Brieger P Laux G Bender W Heuser I Zeiler J Gaebel W Mayr A Möller HJ Riedel M Does early improvement triggered by antidepressants predict response/remission? Analysis of data from a naturalistic study on a large sample of inpatients with major depression Journal of Affective Disorders 2009 115 439 449 10.1016/j.jad.2008.10.011 19027961

[B18] Hennings JM Owashi T Binder EB Horstmann S Menke A Kloiber S Dose T Wollweber B Spieler D Messer T Lutz R Kunzel H Bierner T Pollmacher T Pfister H Nickel T Sonntag A Uhr M Ising M Holsboer F Lucae S Clinical characteristics and treatment outcome in a representative sample of depressed inpatients. Findings from the Munich Antidepressant Response Signature (MARS) project Journal of Psychiatric Research 2008 43 215 229 10.1016/j.jpsychires.2008.05.002 18586274

[B19] Papakostas GI Perlis RH Scalia MJ Petersen TJ Fava M A meta-analysis of early sustained response rates between antidepressants and placebo for the treatment of major depressive disorder Journal of Clinical Psychopharmacology 2006 26 56 60 10.1097/01.jcp.0000195042.62724.76 16415707

[B20] Posternak MA Zimmerman M Is there a delay in the antidepressant effect? A meta-analysis Journal of Clinical Psychiatry 2005 66 148 158 10.4088/JCP.v66n0201 15704999

[B21] Taylor MJ Freemantle N Geddes JR Bhagwagar Z Early onset of selective serotonin reuptake inhibitor antidepressant action: systematic review and meta-analysis Archives of General Psychiatry 2006 63 1217 1223 10.1001/archpsyc.63.11.1217 17088502 PMC2211759

[B22] Stassen HH Angst J Hell D Scharfetter C Szegedi A Is there a common resilience mechanism underlying antidepressant drug response? Evidence from 2848 patients Journal of Clinical Psychiatry 2007 68 1195 1205 10.4088/JCP.v68n0805 17854243

[B23] Szegedi A Müller MJ Anghelescu I Klawe C Kohnen R Benkert O Early improvement under mirtazapine and paroxetine predicts later stable response and remission with high sensitivity in patients with major depression Journal of Clinical Psychiatry 2003 64 413 420 10.4088/JCP.v64n0410 12716243

[B24] Szegedi A Jansen WT van Willigenburg AP van der Meulen E Stassen HH Thase ME Early improvement as a predictor of treatment outcome in patients with major depressive disorder: Why the first 2 weeks really matter-evidence from 6,562 patients Journal of Clinical Psychiatry 2009 70 344 353 10.4088/JCP.07m03780 19254516

[B25] Tadić A Helmreich I Mergl R Hautzinger M Henkel V Hegerl U Early improvement is a predictor of treatment outcome in patients with mild major, minor or subsyndromal depression Journal of Affective Disorders 2010 120 86 93 19428118 10.1016/j.jad.2009.04.014

[B26] Leuchter AF Cook IA Hunter AM Korb AS A new paradigm fort he prediction of antidepressant treatment response Dialogues in Clinical Neuroscience 2009 11 435 446 20135901 10.31887/DCNS.2009.11.4/afleuchter PMC3181929

[B27] Mössner R Mikova O Koutsilieri E Saoud M Ehlis AC Müller N Fallgatter AJ Riederer P Consensus paper of the WFSBP Task Force on Biological Markers: biological markers in depression World J Biol Psychiatry 2007 8 141 174 17654407 10.1080/15622970701263303

[B28] Holsboer F How can we realize the promise of personalized antidepressant medicines? Nature Reviews Neuroscience 2008 9 638 646 10.1038/nrn2453 18628772

[B29] Domenici E Wille' DR Tozzi F Prokopenko I Miller S McKeown S Brittain C Rujescu D Giegling I Turck CW Holsboer F Bullmore ET Middleton F Merlo-Pich E Alexander RC Muglia P Plasma Protein Biomarkers for Depression and Schizophrenia by Multi Analyte Profiling of Case-Control Collections PLoS ONE 2010 5 e9166 10.1371/journal.pone.0009166 20161799 PMC2820097

[B30] Beblo T Herrmann M Neuropsychologische Defizite bei depressiven Störungen Fortschritt Neurologie Psychiatrie 2000 68 1 11 10.1055/s-2000-11640 10705569

[B31] Beblo T Lautenbacher S, Gauggel S (Hrsg.) Neuropsychologie affektiver Störungen 2004 Neuropsychologie psychischer Störungen. Heidelberg: Springer Verlag

[B32] Fossati P Ergis AM Allilaire JF Executive functioning in unipolar depression: a review Encephalopathy 2005 28 97 107 11972136

[B33] Sobin C Sackeim HA Psychomotor symptoms of depression American Journal of Psychiatry 1997 154 4 17 8988952 10.1176/ajp.154.1.4

[B34] Christensen H Griffiths K Mackinnon A Jacomb P A quantitative review of cognitive deficits in depression and Alzheimer-type dementia Journal of the International Neuropsychological Society 1997 3 631 651 9448376

[B35] Veiel HO A preliminary profile of neuropsychological deficits associated with major depression Journal of Clinical and Experimenal Neuropsychology 1997 19 587 603 10.1080/01688639708403745 9342691

[B36] Savard RJ Rey AC Post RM Halstead-Reitan Category Test in bipolar and unipolar affective disorders. Relationship to age and phase of illness Journal of Nervous and Mental Disease 1980 168 297 304 10.1097/00005053-198005000-00010 7365494

[B37] Beblo T Baumann B Bogerts B Wallesch C Herrmann M Neuropsychological Correlates of Major Depression: A Short-term Follow-up Cognitive Neuropsychiatry 1999 4 333 341 10.1080/135468099395864

[B38] Potter GC Kittinger JD Wagner HR Steffens DC Krishnan RR Prefrontal neuropsychological predictors of treatment remission in late life depression Neuropsychopharmacology 2004 29 2266 2271 10.1038/sj.npp.1300551 15340392

[B39] De Groth MH Nolen WA Huijsman AM Bouvy PF Lateralized neuropsychological functioning in depressive patients before and after drug therapy Biological Psychiatry 1996 40 1282 1287 10.1016/0006-3223(95)00654-0 8959293

[B40] Paelecke-Habermann Y Pohl J Leplow B Attention and executive functions in remitted major depression patients Journal of Affective Disorders 2005 89 125 135 10.1016/j.jad.2005.09.006 16324752

[B41] Weiland-Fiedler P Erickson K Waldeck T Luckenbaugh DA Pike D Bonne O Charney DS Neumeister A Evidence for continuing neuropsychological impairments in depression Journal of Affective Disorders 2004 82 253 258 10.1016/j.jad.2003.10.009 15488254

[B42] Nakano Y Baba H Maeshima H Kitajima A Sakai Y Baba K Suzuki T Mimura M Arai H Executive dysfunction in medicated, remitted state of major depression Journal of Affective Disorders 2008 111 46 51 10.1016/j.jad.2008.01.027 18304646

[B43] Neu P Bajbouj M Schilling A Godemann F Berman RM Schlattmann P Cognitive function over the treatment course of depression in middle-aged patients: correlation with brain MRI signal hyper intensities Journal of Psychiatric Research 2005 39 129 135 10.1016/j.jpsychires.2004.06.004 15589560

[B44] Paradiso S Lamberty GJ Garvey MJ Robinson RG Cognitive impairment in the euthymic phase of chronic unipolar depression Journal of Nervous and Mental Disease 1997 185 748 754 10.1097/00005053-199712000-00005 9442186

[B45] Wong JL Wetterneck C Klein A Effects of depressed mood on verbal memory performance versus self-reports of cognitive difficulties International Journal of Rehabilitation and Health 2000 5 85 97 10.1023/A:1012902121486

[B46] Gorlyn M Keilp JG Grunebaum MF Taylor BP Oquendo MA Bruder GE Stewart JW Zalsman G Mann JJ Neuropsychological characteristics as predictors of SSRI treatment response in depressed subjects Journal of Neural Transmission 2008 115 1213 1219 10.1007/s00702-008-0084-x 18629432 PMC3767987

[B47] Mayberg HS Brannan SK Tekell JL Silva JA Mahurin RK McGinnis S Jerabek PA Regional metabolic effects of Fluoxetine in Major Depression: Serial changes and relationship to clinical response Biological Psychiatry 2000 48 830 843 10.1016/S0006-3223(00)01036-2 11063978

[B48] Mayberg HS Modulating dysfunctional limbic-cortical circuits in depression: towards development of brain-based algorithms for diagnosis and optimised treatment British Medical Bulletin 2003 65 193 207 10.1093/bmb/65.1.193 12697626

[B49] Tadić A Gorbulev S Dahmen N Hiemke C Braus DF Röschke J van Calker D Wachtlin D Kronfeld K Gorbauch T Seibert-Grafe M Lieb K EMC Study Group Rationale and design of the randomised clinical trial comparing early medication change (EMC) strategy with treatment as usual (TAU) in patients with Major Depressive Disorder-the EMC trial Trials 2010 11 21 20187947 10.1186/1745-6215-11-21 PMC2837649

[B50] Wittchen HU Weigel A Pfister H DIA-X-Diagnostisches Expertensystem 1996 Frankfurt: Swets Test Services

[B51] Shehaan DV Lecrubier Y M.I.N.I. International Neuropsychiatric Interview 1998 USA Tampa

[B52] American Psychiatric Association Diagnostic and statistical manual of mental disorders 2004 4 American Psychiatric Press: Washington DC

[B53] Dilling H Mombour W Schmidt MH International Classification of Mental and Behavioral Disorders. ICD-10 chapter V 2010 Huber: Bern, Switzerland

[B54] Wittchen HU Zaudig M Fydrich T SKID-I/-II: Strukturiertes klinisches Interview für DSM-IV 1996 Hogrefe: Göttingen

[B55] Hamilton M A rating scale for depression Journal of Neurology, Neurosurgery, Psychiatry 1960 23 56 62 10.1136/jnnp.23.1.56 PMC495331 14399272

[B56] Rush AJ Gullion CM Basco MR Jarrett RB Trivedi HM The inventory of Depressive Symptomatology (IDS) Psychological Medicine 1986 26 477 486 10.1017/S0033291700035558 8733206

[B57] Bullinger M Kirchberger I Fragebogen zum Gesundheitszustand - SF 12 1998 Göttingen: Hogrefe Verlag

[B58] Lehrl S 1969 Erlangen: Perimed Fachbuch Verlagsgesellschaft mbH Mehrfachwahl-Wortschatz-Intelligenztest (MWT-B)

[B59] Aschenbrenner S Tucha O Lange KW RWT. Regensburger Wortflüssigkeits-Test 2000 Göttingen: Hogrefe

[B60] Reitan RM Trail Making Test - Manual for Administration and Scoring 1979 Tucson: Reitan Neuropsychology Laboratory

[B61] Werheid K Hoppe C Thöne A Müller U Müngersdorf M von Cramon DY The Adaptive Digit Ordering Test clinical application, reliability and validity of a verbal working memory test Archives of Clinical Neuropsychology 2002 17 547 565 14591855

[B62] Ruff R Ruff Figural Fluency Test (RFFT) 1988 San Diego: Neuropsychological resources

[B63] Müller MJ Dragicevic A Standardized rater training for the Hamilton Depression Rating Scale (HAMD-17) in psychiatric novices Journal of Affective Disorders 2003 77 65 69 14550936 10.1016/s0165-0327(02)00097-6

[B64] Wagner S Baskaya Ö Lieb K Tadic A Standardized rater training for the Hamilton Depression Scale (HAMD_17_) and the Inventory of Depressive Symptoms (IDS_30CR_) Psychopathology 2011 44 68 70 10.1159/000318162 21072002

